# Age-specific survival in acute myeloid leukemia in the Nordic countries through a half century

**DOI:** 10.1038/s41408-024-01033-7

**Published:** 2024-03-14

**Authors:** Kari Hemminki, Frantisek Zitricky, Asta Försti, Mika Kontro, Bjorn T. Gjertsen, Marianne Tang Severinsen, Gunnar Juliusson

**Affiliations:** 1 Biomedical Center, Faculty of Medicine in Pilsen, Charles University in Prague, 30605 Pilsen, Czech Republic; 2 Division of Cancer Epidemiology, German Cancer Research Center (DKFZ), Im Neuenheimer Feld 580, D-69120 Heidelberg, Germany; 3 Hopp Children’s Cancer Center (KiTZ), Heidelberg, Germany; 4 Division of Pediatric Neurooncology, German Cancer Research Center (DKFZ), German Cancer Consortium (DKTK), Heidelberg, Germany; 5 Institute for Molecular Medicine Finland (FIMM), HiLIFE, University of Helsinki, Helsinki, Finland; 6 Foundation for the Finnish Cancer Institute, Helsinki, Finland; 7 Department of Hematology, Helsinki University Hospital Comprehensive Cancer Center, Helsinki, Finland; 8 Centre for Cancer Biomarkers (CCBIO), Department of Clinical Science, University of Bergen, Bergen, Norway; 9 Hematology Section, Department of Medicine, Haukeland University Hospital, Helse Bergen HF, Bergen, Norway; 10 Department of Hematology, Clinical Cancer Research Unit, Aalborg University Hospital, Aalborg, Denmark; 11 Department of Clinical Medicine, Aalborg University, Aalborg, Denmark; 12 Department of Hematology, Skåne University Hospital, Lund, Sweden; 13 Department of HematologyStem Cell Center, Department of Laboratory Medicine, Lund University, Lund, Sweden

**Keywords:** Epidemiology, Cancer

Dear Editor,

Overall survival has developed well in hematological malignancies but among the main entities, 5-year survival in acute myeloid leukemia (AML) has remained the lowest of all, mainly because of the poor survival of the old patients (half of patients are 70+ years at diagnosis) [1, 2]. The improvements in AML survival were achieved through traditional intensive chemotherapy with cytosine arabinoside (ara-C) and anthracyclines, and these have remained the mainstay of intensive chemotherapy with curative intent [3]. Hematopoietic stem cell transplantation (HSCT) is commonly included in treatment of fit high risk patients, mostly younger than 70 years [4]. However, the applied age/fitness restriction for intensive chemotherapy excludes old and frail patients for whom hypomethylating agents (decitabine or azacytidine) have been used [4, 5]. In Denmark the use of intensive chemotherapy has declined between 2001and 2016 from 40 to 30% of patient at age 71–75 years and remained at 10% or less in older patients; in the same period, the use of hypomethylating agents increased from 10 towards 30% in patients at age 71–80 years and up to 20% in 80+ patients [6]. Palliative or no treatment was offered to 50% of pateints age 71–75 years and in increasing proportions for older patients. The Swedish national guidelines of year 2005 recommended a more intensive initial treatment with ara-C and anthracycline than the common and universally used ‘3 + 7’ regime for patients up to 80 years, whereas low-intensity treatment with hypomethylating agents even for the oldest patients was introduced in 2015 reducing the share for palliative care [7, 8].

Mechanistic understanding of AML and its molecular characterization have markedly increased and this has been translated into novel diagnostic and risk classification, and further to treatment armamentarium with many approved therapies, such as specific inhibitors of AML molecular pathways [3, 4]. Non-therapy related gains in AML management have been achieved through enhanced prognostic tools, refined risk assessment, including estimation of measurable residual disease, and improved supportive care including transfusions and prophylaxis and treatment of infections [3, 4]. The 2022 update of the European LeukemiaNet diagnostic and management recommendations for AML are a synthesis of the new developments introducing genetic aberrations as disease defining features [3]. Intensive chemotherapy is complemented with inhibitors targeting specific mutations, such as *FLT3, IDH1* and *IDH2*, and the mutational profile is now a key component in risk classification [3]. For old, unfit and relapsing patients the *BCL2*-inhibitor venetoclax has recently increased the treatment options [3, 4, 9]. For AML it was approved in Europe in 2021 in combination with a hypomethylating agent. In USA this treatment has shown improved survival in the elderly patients [9]. How the novel molecular medicine will translate to population-level survival figures for AML will be seen in the near future.

We analyzed here survival in AML using the up-to-date NORDCAN database (https://nordcan.iarc.fr/en/database#bloc2), which uses ‘hybrid survival’ methods with an aim to document the most recent survival events. Furthermore, the last covered year is 2021 and thus the data are as recent as any nation-wide cancer registry can deliver. Our specific aim is to analyze trends in age-specific survival in AML through 50 years from the cancer registries of Denmark (DK), Finland (FI), Norway (NO) and Sweden (SE) which supplied the data to NORDCAN [10]. In addition to the standard 1- and 5-year relative survival we developed 5/1-year conditional relative survival to indicate survival for those who survived year 1 to survive additional 4 years. We try to identify the periods and reasons when survival has advanced in various age-groups [11]. We compare the Nordic survival data with the US data. Methods are described in the supplement. Patient numbers by sex, age, period and country are shown in Supplementary Table 1.

Graphical age-specific relative survival in AML for SE patients is described in Fig. 1. We show male data on top for 1- 5/1- and 5-year relative survival (panel A–C) and female data in bottom (panels D–F). The panels follow survival in time sequence from 0 to 1 year, from 1 to 5 year and finally collectively at year 5. Survival in AML in SE improved in all age-groups but the 80–89-year-old for whom many missing data points excluded a proper modeling. A clear improvement in 5/1-year survival implied that survival increased for those that had survived the first year. Five-year survival was equal in men and women with final survival figures of 80% for the youngest patients, decreasing stepwise in 10-year age groups to 70, 45, and 20%. As survival preferentially improved in younger pateints the age-related survival gap widened over the years.

**Fig. 1:**
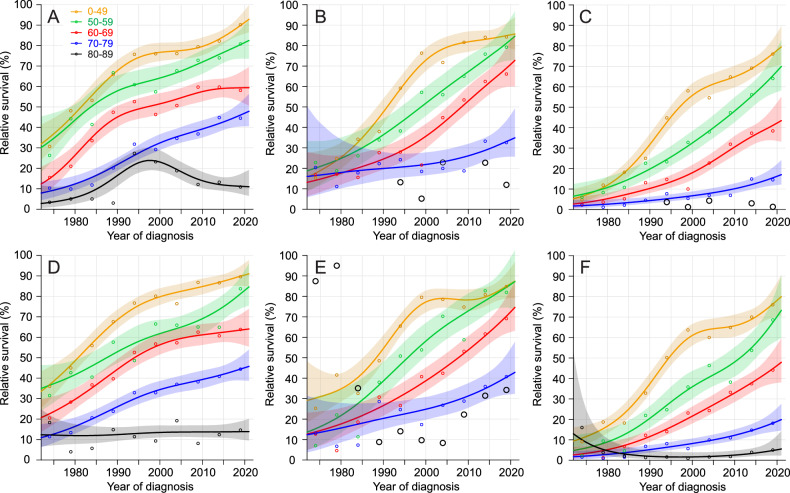
Age-specific relative survival in AML In Sweden. The panels show relative survival in Swedish men (**A**–**C**) and women (**D**–**F**) specifying 1-year (**A,** **D**), 5/1-year (**B**, **E**) and 5-year (**C**, **F**) survival. Shading shows 95%CIs. For the oldest patient data are incomplete and individual data points are shown with large circles.

DK survival deviated from the SE one mostly through weaker 5/1-year survival, particularly for the oldest age-groups (Supplementary Fig. 1). As a result, 5-year survival was below SE results in most age-groups. For FI after year 1, all age-groups but the youngest down-performed SE survival (Supplementary Fig. 2). NO survival was at the level of SE, except for a weak improvement among the 70–79-year-old (Supplementary Fig. 3).

We compared the first (1972–76) and the last (2017–21) 5-year relative survival figures in the Nordic counties in Table 1. In the early period 5-year survival in the young patients was less than 10% compared to the last period of 60–80%; the 50-year increase was around 70% units for most countries. The improvement between the two periods decreased successively in each older age-group, from around 50%, to 30–40% and 19% in age-group 70-79 years. Among 80–89-year-old the last survival figures ranged from 0 to 5%. The best male survival in the last period (underlining) was reached by FI and SE in two and NO in one age-group. SE dominated in female survival with the best data in 4 and NO in one age-group. Notably, SE survival for 70-79-year-old patients was by far the best (the male differences were significant, non-overlapping 95%CIs to other male data), in line with active treatment of 70+ patients since 2005.

**Table 1:** Five-year relative survival in AML in the Nordic countries in 1972–76 and 2017–21.

Period	Denmark	Finland	Norway	Sweden	Denmark	Finland	Norway	Sweden
Male 5-y survival among 0–49 y old					Female 5-y survival among 0-49 y old			
1972–1976	9.6 [5.0–18.5]	4.3 [1.8–10.2]	7.0 [3.0–16.3]	4.7 [2.0–11.1]	9.3 [4.6–18.8]	5.9 [2.5–13.7]	8.3 [4.1–16.8]	9.1 [4.7–17.5]
2017–2021	57.9 [47.8–70.1]	79.3 [70.2–89.5]	78.0 [69.2–88.1]	76.0 [69.2–83.5]	78.7 [70.3–88.0]	72.2 [61.9–84.2]	81.7 [74.4–89.9]	76.0 [69.0–83.7]
Improvement	48.3	75	71	71.3	69.4	66.3	73.4	66.9
Male 5-y survival among 50–59 y old					Female 5-y survival among 50–59 y old			
1972–1976	2.5 [0.4–16.5]	*5.4*	2.8 [0.4–18.1]	6.0 [2.0–17.9]	14.0 [7.0–27.9]	7.2 [2.5–21.4]	3.3 [0.6–19.1]	2.2 [0.3–14.5]
2017–2021	57.5 [45.2–73.1]	34.4 [22.4–52.7]	61.5 [47.8–79.0]	64.0 [54.8–74.7]	45.8 [34.2–61.3]	59.3 [46.1–76.2]	61.4 [47.3–79.6]	68.6 [57.7–81.6]
Improvement	55	*29*	58.7	58.0	31.8	52.1	58.1	66.4
Male 5-y survival among 60–69 y old					Female 5-y survival among 60–69 y old			
1972–1976	5.0 [1.6–15.2]	*5.2*	4.8 [1.6–14.4]	2.6 [0.7–10.3]	4.5 [1.5–13.5]	4.6 [1.5–13.9]	*1.7*	2.6 [0.7–10.1]
2017–2021	34.5 [25.7–46.3]	29.3 [20.7–41.4]	47.2 [37.7–59.0]	38.4 [31.3–47.0]	37.3 [28.1–49.6]	33.2 [24.1–45.6]	37.4 [26.7–52.4]	44.1 [36.3–53.5]
Improvement	29.5	*24.1*	42.4	35.8	32.8	28.6	*35.7*	41.5
Male 5-y survival among 70–79 y old					Female 5-y survival among 70–79 y old			
1972–1976	4.9 [1.4–17.8]	*0.3*	1.9 [0.3–11.5]	2.1 [0.2–13.0]	*3.5*	2.1 [0.3–14.0]	*4.1*	1.5 [0.3–14.5]
2017–2021	9.0 [5.1–15.8]	4.9 [2.5–9.7]	8.3 [4.1–16.9]	14.5]10.3–20.6]	15.6 [9.5–25.5]	12.3 [7.4–20.7]	11.1 [6.0–20.5]	18.1 [13.2–24.8]
Improvement	4.1	*4.6*	6.4	12.4	*12.1*	10.2	*7*	16.6
Male 5-y survival among 80–89 y old					Female 5-y survival among 80–89 y old			
1972–1976	*0.6*	..	..	..	..	..	*3.9*	16.0 [4.7–54.5]
2017–2021	4.2 [0.6–27.2]	5.3 [1.9–14.5]	…	1.3 [0.3–5.5]	1.7 [0.2–13.5]	0.2 [0–14.0]	..	5.0 [2.2–11.4]
Improvement	*3.6*	..	..	..	..	..	..	−11

Male survival is shown on the left coulmns and female survival on the right columns.

In the case of missing data for early period, estimates from subsequent period (1977–81) were used (italics). The best survival figures in the last period are underlined.

Improvement shows the difference between the two periods in % units.

Similar data for 1-year survival are presented in Supplementary Table 2. It is noteworthy that 1-year survival in patients diagnosed before age 50 years reached 90% and successively decreased in older age groups, reaching 40% survival among 70–79-year-old and only 10–20% among 80–89-year-old.

According to NORDCAN, overall male 5-year survival in 2017–21 was 29.3% (95% CI: 25.3–33.9%) in DK and 34.8% (31.7–38.1%) in SE; the comparable female data were 32.1% (27.9–36.8%) and 38.7% (35.2–42.5%) (data for FI and NO were missing). In the US SEER database the 5-year survival figures for AML in 2015–19 were 31.7% for men and 31.9% for women. Age-specific data were available in three age groups: below 50, 50–64 and 65+years. For men the related survival figures were 67.9, 39.4 and 12.3%; for women they were 65.8, 36.6 and 9.0%.

Considering the possible survival advantage of improved management one has to first consider the target population and its size. For AML about 50% (less in the early period) of patients are diagnosed at age over 69 years, at which age many patients were unlikely candidates for intensive chemotherapy, except in SE [8, 12]. The new therapies introduced during the past years have been restricted to smaller subsets of patients and are mostly not available outside clinical studies, except for midostaurin (about 2018), gemtuzumab and venetoclax (SE 2021, DK and FI 2022, NO 2023) [13]. Survival of patients older than 69 years has historically been poor and any recent improvement should show in the data presented. According to Table 1 (and all figures) the good news was that for 70-70-year-old 1-year survival increased from 10 to over 40% (less in NO) which may suggest the impact of the more active hypomethylating therapy [4, 6]. In this age group also 5-year survival increased, most (15–18% units) for SE men and women. The SE advantage could be seen in the conditional 5/1-year survival which indicated that survival clearly increased between years 1 and 5 (Fig. 1). This SE experience for the 70–79-year old patients may suggest that the treatment guidelines of 2005 recommending an intensive initial treatment with ara-C and anthracycline may have contributed to the positive results [7, 8].

We discuss limitations of the study in the supplement; these include no possibility to distinguish childhood AML (25% of patients below 50 years) or individual subtypes of AML.

In conclusion, this study demonstrates a steady increase in AML survival in all but the 80–89-year-old pateints. According to SE and DK experience, survival gains were accomplished through more intensive therapy, novel agents (yet many of them were introduced only recently), extended use of HSCT, improved supportive care and overall population health [6, 8, 14]. The preferential survival improvements in young patients lead to widening of the age-related survival gap. The main concern is the over 80-year-old population which is increasing to one quarter of all patients. The newly approved upfront venetoclax-hypomethylating agent combination is likely to help improve survival among patients over 75 years or those with comorbidities, waiting population-level verification in future survival studies.

### Supplementary information

Supplementary material

Supplementary figures

## Data Availability

Publicly available data were used from the NORDCAN database.
