# Supplementary material for: Age-specific survival in acute myeloid leukemia in the Nordic countries through a half century
Source: Blood Cancer J. 2024 Mar 14;14(1):44. doi: 10.1038/s41408-024-01033-7 (PMC10937905; doi:10.1038/s41408-024-01033-7)
Supplement: Supplementary file 1 — Supplementary material [file 41408_2024_1033_MOESM1_ESM.docx]

AGE-SPECIFIC SURVIVAL IN ACUTE MYELOID LEUKEMIA IN THE NORDIC COUNTRIES THROUGH A HALF CENTURY

Words: 1725 (text).

Survival in old acute myeloid leukemia (AML) patients has been poor. However novel therapies have been introduced and we aim to analyze whether the most up-to-date age-group specific survival figures show improvements. The NORDCAN database of the Danish (DK), Finnish (FI), Norwegian (NO) and Swedish (SE) cancer registries was used to analyze 1- and 5- year and 5/1-years conditional relative survival through years 1972-2021.

METHODS

The data originate from the NORDCAN database which was accessed at the IARC website (<https://nordcan.iarc.fr/en/database#bloc2>) in the fall of 2023/winter 2024 (1-3). The disease codes as well as well as included age groups (0-89 years) were fixed in the database. The proportion of patients diagnosed below age 20 was between 4 and 5% in the last period but they accounted for 25% of patients in age group below 50 years.

The International Classification of Diseases (ICD) version 10 codes for AML were C92.0 (acute myeloid leukemia), C92.3-C92.6 (myelosarcoma, acute promyelocytic leukemia, acute myelocytic leukemia and AML with 11q23). Patient data were provided by individual cancer registries and some variation was likely as to how they translated the earlier codes to ICD-10. Also the individual leukemia types were heterogeneous and IARC call the entity as ‘acute myeloid leukemias’. The AML codes were selected by the Nordic cancer registries with more than 60 years of collaboration and IARC acknowledged high quality already decades ago (4). An important criteria for the selection of the codes was that they were used similarly in the four countries. The NORDCAN code selection did not include acute myeloid leukemia with multilineage dysplasia, other myeloid leukemia and myeloid leukemia, unspecified. The two latter ones were probably excluded because of non-specificity and the first one because of non-uniform registration between the countries. The incidence of AML in Europe was 3.62/100,000 according to a publication from 2010 (5). The incidence of acute myeloid leukemia with multilineage dysplasia was 1.7% of all AML, so it was very rare in that study (5). Acute promyelocytic leukemia is a distinct AML subtype which was included among AML in NORDCAN. According to a SE study it accounted for 3.2% of all AML (incidence 0.15/100,000) with a median age of 54 years and 39% mortality within 30 days (6).

Age-specific survival data were available from 1972 through 2021 and the analysis was based on the cohort survival method for periods from 1972–2016 and a hybrid analysis combining period and cohort survival in the last period 2017-2021. Age group specific relative survival was estimated using the Pohar Perme estimator (7). National general population life-tables stratified by sex, year and age were used in the calculation of expected survival. Death certificate only cases were not included. Patients 90 years or older were excluded. Groups were analyzed if minimum 30 patients were alive at start and with minimum 3 patients in any one of age-groups used for weights. Significant differences were called when 95% confidence intervals (CIs) were non-overlapping.

The temporal trends in relative survival were modelled using Bayesian generalized additive models (with Gaussian link function) (8). The modelling was performed on cumulative hazard scale, which allowed to model input uncertainty based on asymmetric CIs provided by NORDCAN. Point estimates in the middle of the 5-year periods were assinged as an input for the model of temporal trends and for visualization purposes. In case of missing data for 80-89-year-old patients, not allowing full modeling, only available relative survival estimates for patients diagnosed in respective 5-year period are shown.

Survival data from USA was accessed at the US Surveillance, Epidemiology and End Results (SEER) web site for years 2015–19 for Whites and Hispanics (https://seer.cancer.gov/statistics-network/explorer/application.html?site=1&data_type=1&graph_type=2&compareBy=sex&chk_sex_3=3&chk_sex_2=2&rate_type=2&race=1&age_range=1&hdn_stage=101&advopt_precision=1&advopt_show_ci=on&hdn_view=0&advopt_display=2#graphArea).

RESULTS

The NORDCAN database was used for the period 1972 to 2021 and the distribution of patients is shown in **Supplementary Table 1**. In the first 10-year period (1972-81) the oldest AML patients (80-89 years) accounted for 12% of all male patients (FI 6%) and 12% of female patients (NO 16%). In the last 10 years (2012-21), the oldest men accounted for 21% all patients. Among women the proportions were for FI 28%, SE 25% and 22% for DK and NO. The combined 70+ year population accounted for 50% of all AML patients in the last period.

We compared the first (1972-76) and the last (2017-21) 1- year relative survival figures in each of the 5 age-groups in **Supplementary Table 2**. In the early period 1-year survival for the 0-49-year-old men and women ranged between 30 and 47% and it increased in 50 years to 80-91%, or as much as 60 % units for FI patients and SE men. The 50-year increase in survival decreased in each older age-group. For the 70-79-year-old the increase was still some 30 % points but for the 80-89-year-old population the starting level of survival was about 10% and it increased was uneven between the countries; 1.year survival was about 20% in DK and FI and less in NO and SE. The best male 1-year survival figures in the last period were for NO and SE in two and DK in one age-group; for female DK and FI scored two and SE one best ranks.

Graphical age-specific relative survival in AML for DK patients is described in **Supplementary Fig. 1.** Male data are shown on top and female data in bottom sequentially for 1- 5/1- and 5-year relative survival. DK male and female 1-year survival (panels A and D) increased well through the 50 years with a diagnostic age gradient spanning from about 90% for patients diagnosed before age 50 years to 45% among 70-79-year-old. Survival for 80-89-year-old improved only marginally and remained below 20%. Conditional 5/1-year survival (panels B and E) showed also increasing trends but some 10 % units below those for 1-year survival; the exceptions were male survival for age below 50 year which culminated in 2010 (with broad 95%CIs) and survival for 60-69-year-old which remained at the level of 1-year survival. Because of low case numbers for 80-89-year-old, only some data points could be shown. 5-year survival (panels C and F) was a sum of the previous panels which was reflected by the shapes of the curves. The final 5-year survival for women below age 50 years was over 80%, compared to male below 60%. In the next age group male survival was over 60%, 10% unit better than female survival. In other age groups no large differences were noted and for 70-79%-year-old survival remained below 20%.

Survival in AML for FI patients is similarly described in **Supplementary Fig. 2.** The main difference to the DK 1-year survival was very strong early development and accordingly by 1990 all age-specific survival figures exceeded the DK ones. Since then the FI tempo slowed down, particularly for 50-69-year-old men whose final survival was below the DK level. FI 80-89-year-old reached a 1-year survival of over 20%. FI 5/1-year survival developed well particularly for the youngest men but not for 70-79-year-old whose survival decreased to less than 20%. Female 5/1-year survival did not improve after year 2000, except for 50-59-year-old. The final 5-year survival was over 80% for men below 50 years of age and it was below 40% for older men but below 10% for 70-79-year old. Female 5-year survival was better than male survival except for the youngest patients.

In NO 1-year survival was similar to FI but 5/1-year survival improved better than the FI one resulting in 5-year survival better than in FI (**Supplementary Fig. 3**). The exception was survival for the youngest men (80% in both countries). The final 5-year survival was at 11% for 80-89-year-old women.

LIMITATIONS AND CONCLUSIONS

The main limitation of the study is that NORDCAN contains no data on clinical presentation or treatment. Another limitation was that we were not able to distinguish childhood AML with a relatively good prognosis. It was estimated to account for 25% of cases in patients below 50 years of age. The third weakness that we could not distinguish individual subtypes of AML as this option is not available in NORDCAN. In long-term studies, a question may be if the patient population has remained homogeneous over the study. We noted that the proportion of 80-89-year old patients had increased markedly over time. This may be related to the aging of the population with a generally better health status but increased diagnostic activity in the old population may be another explanation, as has been suggested in studies on multiple myeloma (8). WHO disease classification for AML has changed in 2001 and 2016 but these have apparently not influenced overall incidence trends for AML in the Nordic countries (<https://nordcan.iarc.fr/en/database#bloc2>). AML is a rare malignancy and comparison of specific age groups in periods compromises statistical power and 95%CIs are wide. A further limitation is the generalizability of the results, originating from the four Nordic countries with over 25 million inhabitants. Treatment may be further modified and more advanced in other settings (and certainly varies within any individual Nordic country). However these are the most up-to-date survival figures for the entire population.

For outside Nordic comparisons, 5-year survival data for year 2013-18 from the Netherlands for patients over 60 years was reported in age-groups: age 60-64 years 30%, 65-69 years 21%, 70-74 years 10%, 75-79 years 3% and over 80 years 2% (9). Based on the present data, SE 5-year survival figures for 2012-2016 for combined sexes were about 38% for 60-69-year-old, 15% for 70-79 year old and 4% for 80-89 year old. These were better than the Dutch figures. The overall Nordic male 5-year survival figures in 2017-21 ranged between FI 25.5% and in SE 34.8%; for women the available figures ranged from DK 32.1% and SE 38.7%. These can be compared to US male survival of 31.7% for men and 31.9% for women which we collected from the SEER database for years 2015-19. These survival figures were somewhat lower than the Nordic figures. In agreement with the Nordic data, age-group differences were large, and the oldest available patients (65+ years) showed only a 10% survival. This limited international comparison suggests that the Nordic survival data are competitive.

In conclusion, this study demonstrates a steady increase in AML survival in all but the oldest patients. The consequence of the positive development in younger patients was that the negative age-related survival gap widened over the years. Population-level application of the novel therapies is hoped to start closing the gap.

LEGENDS TO FIGURES

**Supplementary Fig. 1.** Age-specific relative survival in AML for Danish men (A-C) and women (D-F) specifying 1-year (A and D), 5/1-year (B and E) and 5-year (C and F) survival. Shading shows 95%CIs. For the oldest patient data are incomplete and individual data points are shown with large circles.

**Supplementary Fig. 2.** Age-specific relative survival in AML for Finnish men (A-C) and women (D-F) specifying 1-year (A and D), 5/1-year (B and E) and 5-year (C and F) survival. Shading shows 95%CIs. For the oldest patient data are incomplete and individual data points are shown with large circles.

**Supplementary Fig. 3.** Age-specific relative survival in AML for Norwegian men (A-C) and women (D-F) specifying 1-year (A and D), 5/1-year (B and E) and 5-year (C and F) survival. Shading shows 95%CIs. For the oldest patient data are incomplete and individual data points are shown with large circles.

REFERENCES

1. Engholm G, Ferlay J, Christensen N, Bray F, Gjerstorff ML, Klint A, et al. NORDCAN--a Nordic tool for cancer information, planning, quality control and research. Acta Oncol. 2010;49(5):725-36.

2. Pukkala E, Engholm G, Hojsgaard Schmidt LK, Storm H, Khan S, Lambe M, et al. Nordic Cancer Registries - an overview of their procedures and data comparability. Acta Oncol. 2018;57:440-55.

3. Larønningen S AG, Bray F, Engholm G, Ervik M, Guðmundsdóttir EM, Gulbrandsen J, Hansen HL, Hansen HM, Johannesen TB, Kristensen S, Kristiansen MF, Lam F, Laversanne M, Miettinen J, Mørch LS, Ólafsdóttir E, Pejicic S, Petterson D, Steig BÁ, Skog A, Tian H, Aagnes B, Storm HH. NORDCAN: Cancer Incidence, Mortality, Prevalence and Survival in the Nordic Countries, Version 9.3 (02.10.2023). 2023.

4. IARC, editor. Cancer Incidence in Five Continents. Lyon: IARC; 2002.

5. Sant M, Allemani C, Tereanu C, De Angelis R, Capocaccia R, Visser O, et al. Incidence of hematologic malignancies in Europe by morphologic subtype: results of the HAEMACARE project. Blood. 2010;116(19):3724-34.

6. Lehmann S, Ravn A, Carlsson L, Antunovic P, Deneberg S, Möllgård L, et al. Continuing high early death rate in acute promyelocytic leukemia: a population-based report from the Swedish Adult Acute Leukemia Registry. Leukemia. 2011;25(7):1128-34.

7. Lundberg FE, Andersson TM, Lambe M, Engholm G, Mørch LS, Johannesen TB, et al. Trends in cancer survival in the Nordic countries 1990-2016: the NORDCAN survival studies. Acta Oncol. 2020;59(11):1266-74.

8. Blimark CH, Vangsted AJ, Klausen TW, Gregersen H, Szabo AG, Hermansen E, et al. Outcome data from >10 000 multiple myeloma patients in the Danish and Swedish national registries. Eur J Haematol. 2022;108(2):99-108.

9. Kaplan ZLR, van Leeuwen N, Posthuma EFM, Visser O, Huls G, van de Loosdrecht AA, et al. Improved relative survival in older patients with acute myeloid leukemia over a 30-year period in the Netherlands: a long haul is needed to change nothing into something. Leukemia. 2022;36(2):596-8.

|  | Denmark | | | | | | | | | |
| --- | --- | --- | --- | --- | --- | --- | --- | --- | --- | --- |
| Age group | 1972-1976 | 1977-1981 | 1982-1986 | 1987-1991 | 1992-1996 | 1997-2001 | 2002-2006 | 2007-2011 | 2012-2016 | 2017-2021 |
| 0-49 | 104/89 | 92/90 | 110/112 | 108/127 | 90/106 | 112/110 | 127/115 | 93/91 | 65/97 | 92/75 |
| 50-59 | 60/68 | 76/72 | 59/51 | 78/52 | 72/53 | 92/66 | 64/73 | 59/61 | 60/60 | 59/55 |
| 60-69 | 99/105 | 131/103 | 151/121 | 136/102 | 106/101 | 113/90 | 113/94 | 139/95 | 114/85 | 102/83 |
| 70-79 | 89/80 | 142/150 | 159/137 | 176/137 | 161/134 | 161/134 | 177/121 | 128/96 | 163/110 | 171/131 |
| 80+ | 32/37 | 66/60 | 57/74 | 66/87 | 89/90 | 100/94 | 100/90 | 123/117 | 104/105 | 124/90 |
|  | Finland | | | | | | | | | |
| Age group | 1972-1976 | 1977-1981 | 1982-1986 | 1987-1991 | 1992-1996 | 1997-2001 | 2002-2006 | 2007-2011 | 2012-2016 | 2017-2021 |
| 0-49 | 120/93 | 87/66 | 83/81 | 95/73 | 86/78 | 85/81 | 69/94 | 62/90 | 75/63 | 68/75 |
| 50-59 | 40/44 | 43/48 | 52/48 | 37/27 | 38/42 | 48/39 | 55/60 | 56/43 | 45/44 | 37/41 |
| 60-69 | 67/74 | 80/70 | 63/65 | 63/79 | 75/82 | 77/76 | 80/68 | 94/80 | 120/104 | 79/63 |
| 70-79 | 51/64 | 79/92 | 73/114 | 78/84 | 87/106 | 118/108 | 138/117 | 132/123 | 151/119 | 151/104 |
| 80+ | 16/21 | 21/43 | 29/46 | 37/43 | 49/58 | 46/88 | 59/99 | 71/106 | 97/115 | 110/117 |
|  | Norway | | | | | | | | | |
| Age group | 1972-1976 | 1977-1981 | 1982-1986 | 1987-1991 | 1992-1996 | 1997-2001 | 2002-2006 | 2007-2011 | 2012-2016 | 2017-2021 |
| 0-49 | 74/85 | 90/69 | 67/59 | 75/64 | 88/75 | 101/78 | 82/84 | 84/77 | 86/88 | 83/95 |
| 50-59 | 41/35 | 45/37 | 44/37 | 34/37 | 34/22 | 52/54 | 57/43 | 68/35 | 46/48 | 52/45 |
| 60-69 | 75/48 | 91/67 | 81/70 | 84/43 | 87/70 | 74/57 | 84/51 | 101/80 | 87/73 | 105/67 |
| 70-79 | 82/80 | 98/99 | 128/98 | 88/87 | 114/87 | 122/94 | 134/90 | 117/83 | 105/74 | 124/114 |
| 80+ | 33/42 | 57/57 | 66/55 | 62/57 | 57/82 | 64/87 | 87/103 | 95/116 | 78/76 | 99/92 |
|  | Sweden | | | | | | | | | |
| Age group | 1972-1976 | 1977-1981 | 1982-1986 | 1987-1991 | 1992-1996 | 1997-2001 | 2002-2006 | 2007-2011 | 2012-2016 | 2017-2021 |
| 0-49 | 117/100 | 134/122 | 132/141 | 140/137 | 136/157 | 138/146 | 125/171 | 156/138 | 159/151 | 129/132 |
| 50-59 | 60/55 | 70/70 | 84/70 | 78/77 | 80/62 | 107/79 | 95/99 | 89/89 | 97/68 | 104/75 |
| 60-69 | 104/100 | 149/95 | 161/167 | 159/143 | 123/153 | 176/148 | 170/127 | 175/131 | 180/152 | 159/126 |
| 70-79 | 102/112 | 144/152 | 221/183 | 223/180 | 234/214 | 243/231 | 244/202 | 199/181 | 215/201 | 259/222 |
| 80+ | 53/44 | 60/75 | 82/112 | 97/127 | 128/147 | 171/196 | 184/218 | 160/189 | 179/209 | 164/169 |

**SUPPLEMENTARY TABLE 1.** Cases numbers diagnosed with AML in Nordic countries in 5-year periods between 1972 and 2021 (N men/N women)

| **SUPPLEMENTARY TABLE 2.** 1-year relative survival in AML in the Nordic countries in 1972-76 and 2017-21. In the case of missing data for early period, estimates from subsequent period (1977-81) are shown (italics). The best survival figure in the last period is underlined. | | | | | | | | |  |
| --- | --- | --- | --- | --- | --- | --- | --- | --- | --- |
|  |  |  |  |  |  |  |  |  |  |
| MALE 1-Y SURVIVAL AMONG 0-49 Y OLD | | |  |  |  | FEMALE 1-Y SURVIVAL AMONG 0-49 Y OLD | | |  |
| Period | Denmark | Finland | Norway | Sweden |  | Denmark | Finland | Norway | Sweden |
| 1972-1976 | 42.5 [33.1-54.4] | 34.3 [26.7-44.1] | 34.4 [25.0-47.2] | 30.7 [23.1-40.8] |  | 47.4 [37.4-60.1] | 31.5 [23.0-43.0] | 41.3 [32.0-53.2] | 36 [27.3-47.5] |
| 2017-2021 | 82.6 [74.9-91.0] | 89.9 [83.0-97.4] | 92.6 [87.1-98.5] | 90.2 [85.3-95.5] |  | 91.3 [85.2-97.7] | 89.7 [82.797.3] | 90.6 [84.9-96.7] | 89.6 [84.6-94.9] |
| Improvement | 40.1 | 56.6 | 58.2 | 59.5 |  | 43.9 | 58.2 | 49.3 | 53.6 |
|  |  |  |  |  |  |  |  |  |  |
| MALE 1-Y SURVIVAL AMONG 50-59 Y OLD | | |  |  |  | FEMALE 1-Y SURVIVAL AMONG 50-59 Y OLD | | |  |
| Period | Denmark | Finland | Norway | Sweden |  | Denmark | Finland | Norway | Sweden |
| 1972-1976 | 27.7 [17.2-44.8] | 10.6 [4.2-26.5] | 27.9 [16.9-46.0] | 26.3 [16.8-41.2] |  | 36.8 [25.8-52.7] | 23.5 [13.7-40.3] | 20.3 [10.6-39.1] | 31.5 [20.7-47.8] |
| 2017-2021 | 77.8 [67.7-89.5] | 56.5 [43.0-74.5] | 76.7 [65.8-89.4] | 80.8 [73.4-88.9] |  | 72 [60.8-85.2] | 76.4 [64.6-90.5] | 78.3 [67.2-91.3] | 83.7 [75.5-92.7] |
| Improvement | 60.1 | 45.9 | 48.8 | 54.5 |  | 35.2 | 52.9 | 50 | 52.2 |
|  |  |  |  |  |  |  |  |  |  |
| MALE 1-Y SURVIVAL AMONG 60-69 Y OLD | | |  |  |  | FEMALE 1-Y SURVIVAL AMONG 60-69 Y OLD | | |  |
| Period | Denmark | Finland | Norway | Sweden |  | Denmark | Finland | Norway | Sweden |
| 1972-1976 | 12.8 [7.0-23.5] | 14.5 [7.4-28.5] | 14 [7.9-24.9] | 15.5 [9.4-25.4] |  | 19.3 [12.1-30.9] | 20.4 [12.8-32.5] | 14.9 [7.5-29.4] | 20.5 [13.4-31.2] |
| 2017-2021 | 61.8 [52.9-72.2] | 55.5 [45.2-68.2] | 63.8 [55.1-73.9] | 58.1 [50.9-66.4] |  | 58.7 [48.7-70.7] | 69.5 [59.0-81.8] | 65 [54.3-77.9] | 63.8 [56.0-72.6] |
| Improvement | 49 | 41 | 49.8 | 42.6 |  | 39.4 | 49.1 | 50.1 | 43.3 |
|  |  |  |  |  |  |  |  |  |  |
| MALE 1-Y SURVIVAL AMONG 70-79 Y OLD | | |  |  |  | FEMALE 1-Y SURVIVAL AMONG 70-79 Y OLD | | |  |
| Period | Denmark | Finland | Norway | Sweden |  | Denmark | Finland | Norway | Sweden |
| 1972-1976 | 19.5 [11.7-32.5] | 7.8 [2.7-22.7] | 1.9 [0.3-11.5] | 10.3 [5.4-19.9] |  | 11.3 [5.4-23.9] | 5.7 [1.9-16.7] | 9.5 [4.7-19.1] | 11.4 [6.4-20.3] |
| 2017-2021 | 43 [36.0-51.4] | 41.3 [34.1-50.0] | 35.6 [27.8-45.6] | 44.5 [38.6-51.3] |  | 45.5 [37.4-55.3] | 42.2 [33.3-53.5] | 31.5 [23.8-41.6] | 44.4 [38.3-51.5] |
| Improvement | 23.5 | 33.5 | 33.7 | 34.2 |  | 34.2 | 36.5 | 22 | 33 |
|  |  |  |  |  |  |  |  |  |  |
| MALE 1-Y SURVIVAL AMONG 80-89 Y OLD | | | | |  | FEMALE 1-Y SURVIVAL AMONG 80-89 Y OLD | | | |
| Period | Denmark | Finland | Norway | Sweden |  | Denmark | Finland | Norway | Sweden |
| 1972-1976 | *16* | .. | 11.3 [3.9-32.6] | 3.4 [0.6-20.1] |  | *10.6* | *3* | 6.1 [1.7-22.5] | 18.3 [8.3-40.2] |
| 2017-2021 | 21.3 [14.3-31.6] | 17.3 [10.8-27.6] | 14.2 [8.0-25.4] | 10.9 [6.8-17.4] |  | 13.5 [7.4-24.7] | 23.6 ]16.0-34.7] | 7.9 [3.1-19.9] | 14.6 [9.7-21.8] |
| Improvement | *5.3* | .. | 2.9 | 7.5 |  | *2.9* | *20.6* | 1.8 | -3.7 |
| Improvement shows the difference between the two periods in % units. | | |  |  |  |  |  |  |  |
