# Supplementary figures and images for: Age-specific survival in acute myeloid leukemia in the Nordic countries through a half century
Source: Blood Cancer J. 2024 Mar 14;14(1):44. doi: 10.1038/s41408-024-01033-7 (PMC10937905; doi:10.1038/s41408-024-01033-7)

Fig. 1

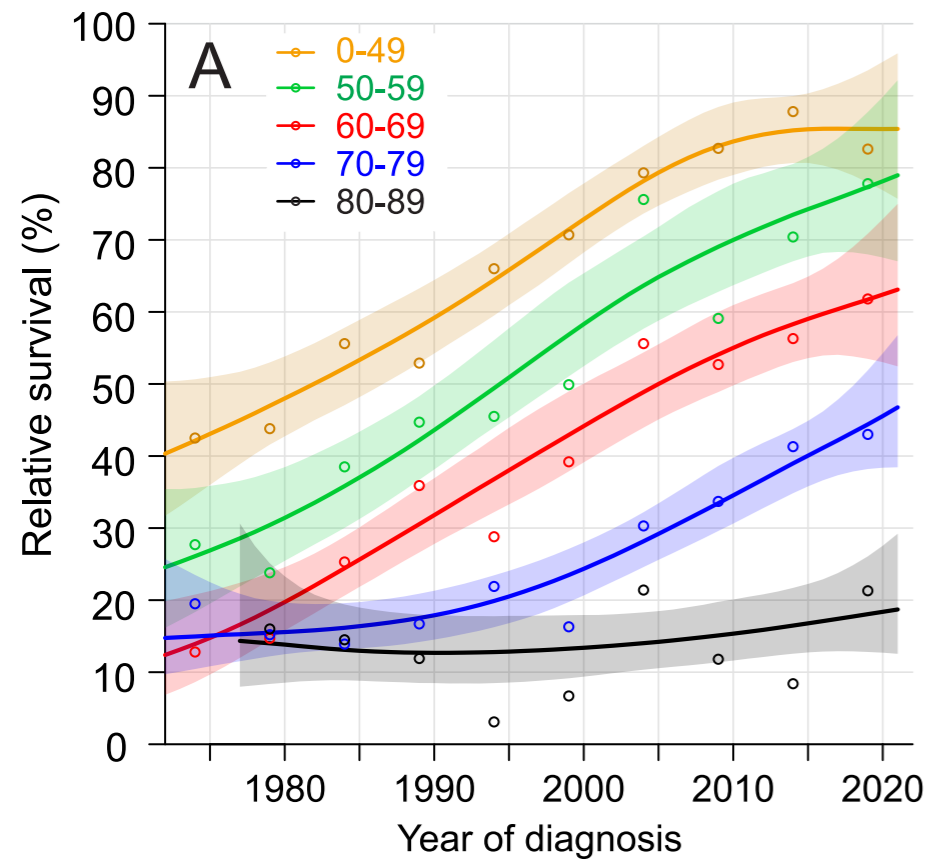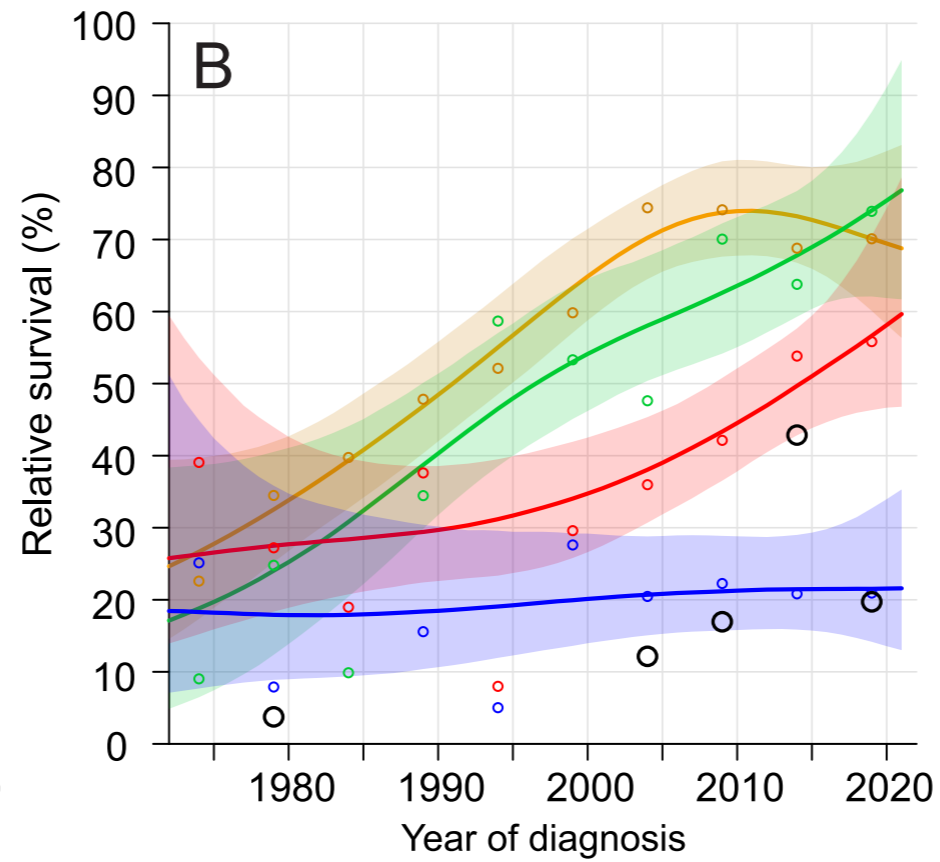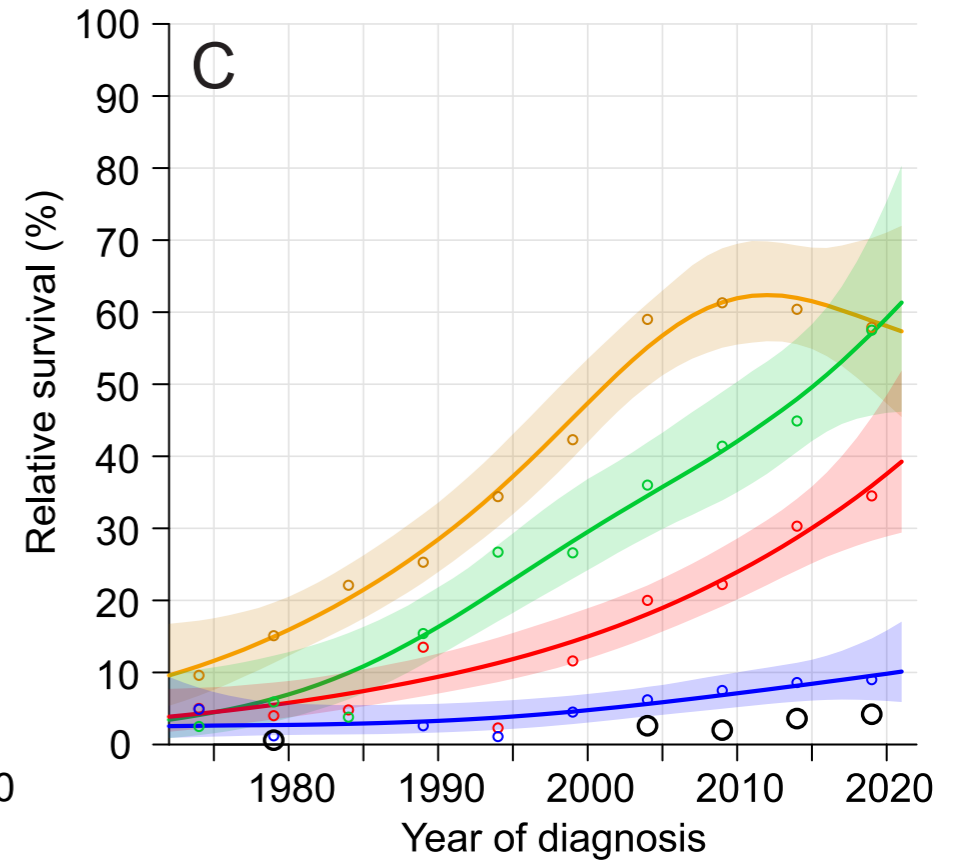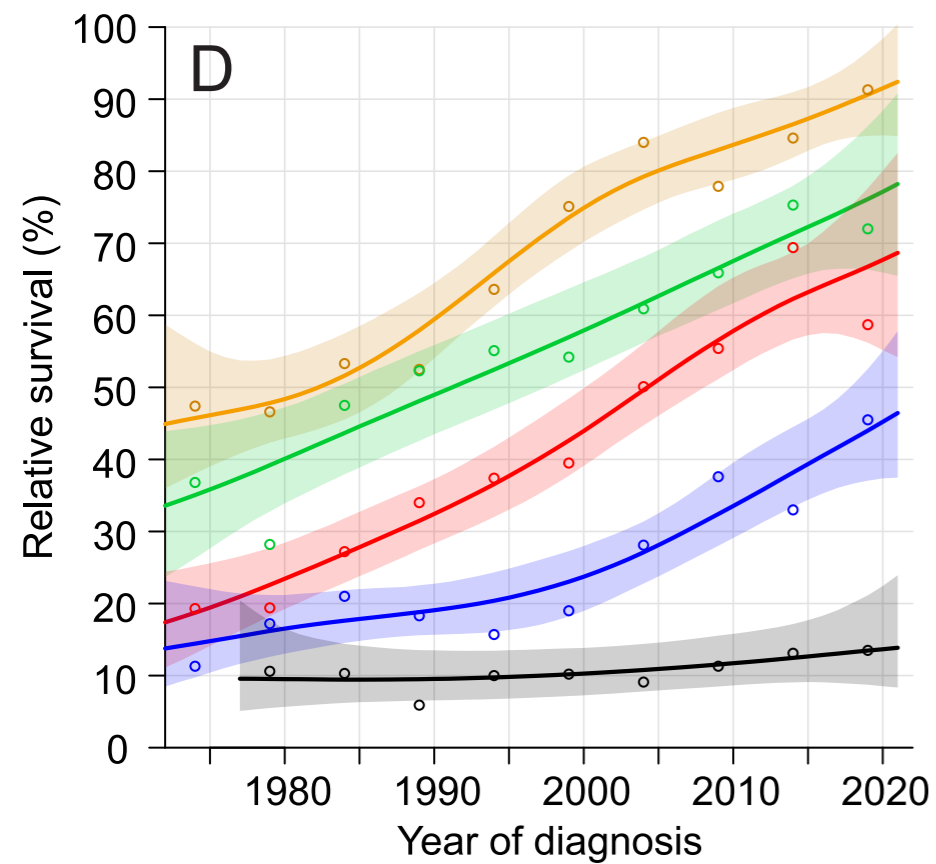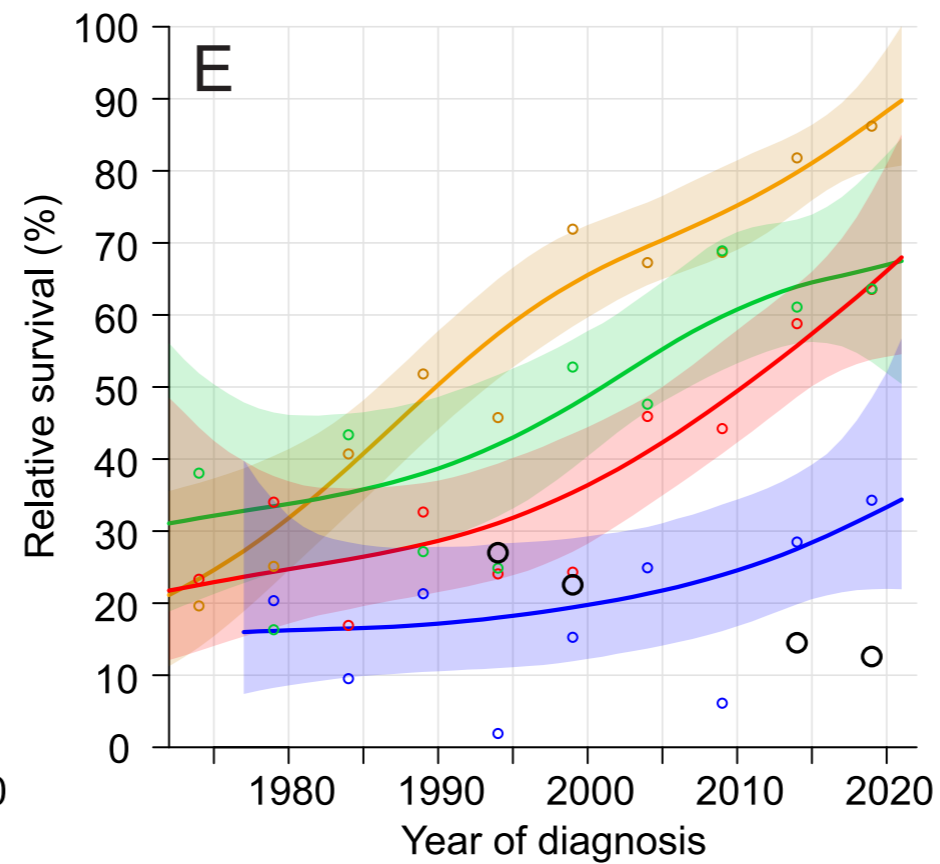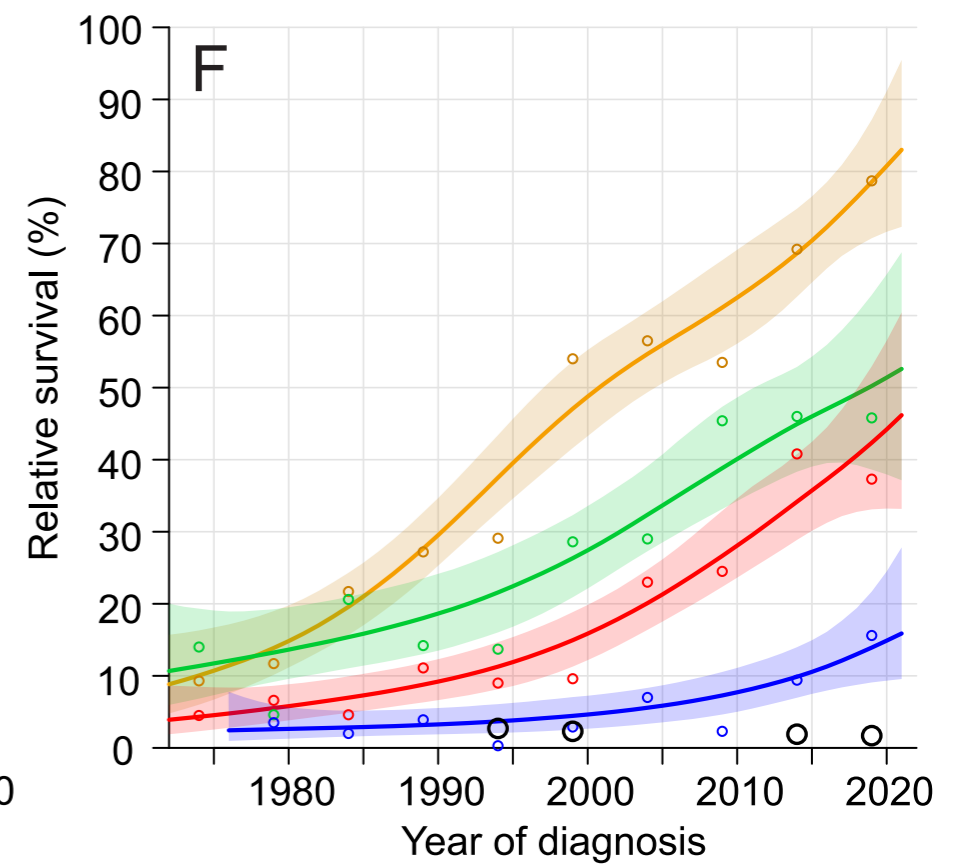

Fig. 2

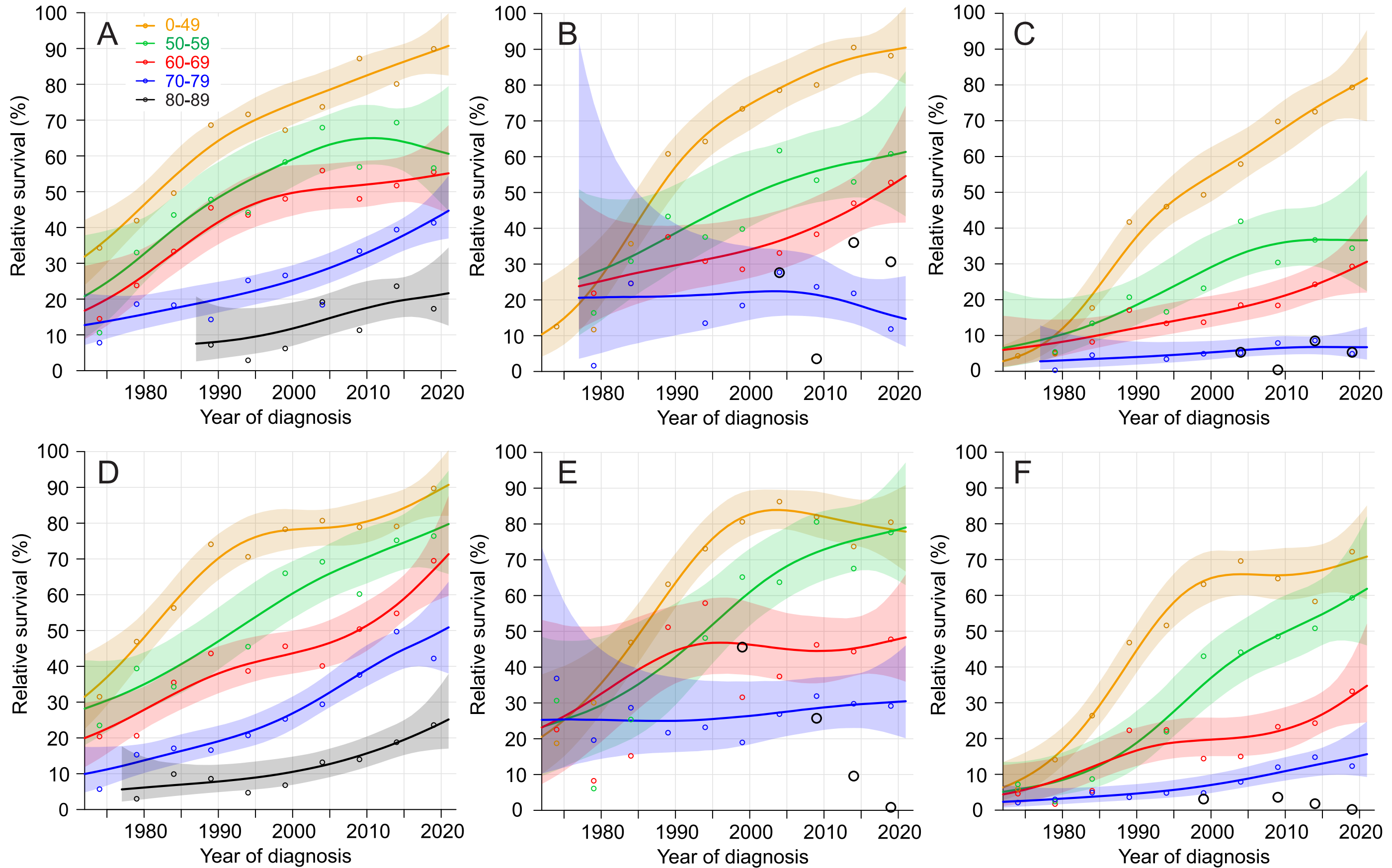

Fig. 3

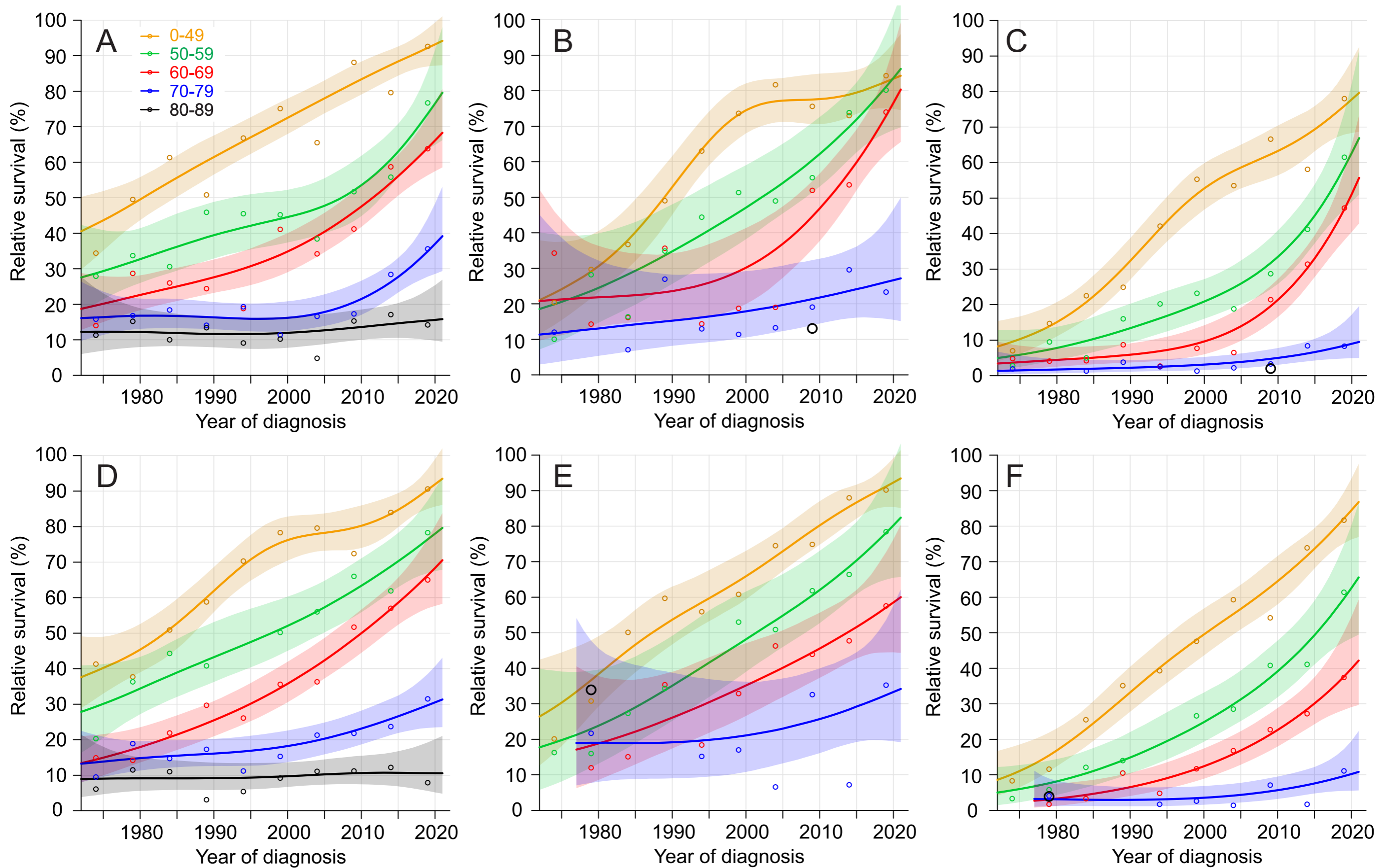

Supplement: Supplementary file 2 — Supplementary figures [file 41408_2024_1033_MOESM2_ESM.pdf]
